# Low Detection Rate of Possible Anesthesia-Related Complications After Pediatric Inguinal Hernia Repair Challenges Current Postoperative Monitoring Protocols

**DOI:** 10.3390/jcm15041639

**Published:** 2026-02-21

**Authors:** Roxanne Eurlings, Nakhari A. S. Alberto, Joep P. M. Derikx, Hamit Cakir, Michiel W. P. de Wolf, Wim G. van Gemert, Ruben G. J. Visschers

**Affiliations:** 1 Department of Pediatric Surgery, MosaKids Children’s Hospital, Maastricht University Medical Center+ (MUMC+), P. Debyelaan 25, 6229 HX Maastricht, The Netherlands; 2 Research Institute for Nutrition and Translational Research in Metabolism NUTRIM, Faculty of Health Medicine and Life Sciences FHML, Maastricht University, Universiteitssingel 40, 6229 ER Maastricht, The Netherlands; 3 Department of Pediatric Surgery, Emma Children’s Hospital, Amsterdam University Medical Center (AUMC), Meibergdreef 9, 1105 AZ Amsterdam, The Netherlands; 4 European Consortium of Pediatric Surgery (MUMC+, Uniklinik Aachen, Centre Hospitalier Chrétien Liège), Maastricht, P. Debyelaan 25, 6229 HX Maastricht, The Netherlands; 5 Department of Anesthesiology and Pain Medicine, Maastricht University Medical Center+ (MUMC+), P. Debyelaan 25, 6229 HX Maastricht, The Netherlands

**Keywords:** inguinal hernia repair, anesthesia, postoperative monitoring, prematurity, respiratory complications, anesthesia-related complications

## Abstract

**Background**: Inguinal hernia repair (IHR) is frequently performed in infants, often under general anesthesia. Preterm infants are routinely monitored for 24 h postoperatively, due to high reported rates of respiratory complications. However, recent data suggest a decline in these events, prompting a reevaluation of the existing monitoring protocols. This study assesses the detection of (possible) anesthesia-related complications within 24 h after IHR in infants under 3 months of age and aims to identify risk factors for these complications. **Methods**: This retrospective cohort study included consecutive patients aged ≤ 3 months who underwent IHR between November 2015 and August 2023. All underwent IHR under general anesthesia. Subjects were compared based on whether they experienced possible anesthesia-related complications within 24 h after surgery or not. A logistic regression model was constructed and the number needed to monitor was calculated. **Results**: 306 patients were included, of which 36.3% were prematurely born (gestational age < 37 weeks) and the mean postconceptional age at surgery was 47.7 ± 4.8 weeks. Possible anesthesia-related complications were detected in 10 patients (3.3%), but only 8 (2.6%) were likely attributable to anesthesia. Events included desaturations, convulsions, fever, and a choking incident. Significant differences were found in patients experiencing complications when they had pre-existing respiratory (*p* = 0.013) or circulatory (*p* = 0.016) comorbidities. The postconceptional age (PCA) and gestational age (GA) were not significantly different between groups. Univariate logistic regression did not show a significant correlation between anesthesia-related complications and PCA or GA. **Conclusions**: Our data corroborates the suggestion that prematurity and PCA alone are not the main characteristics upon which postoperative monitoring protocols should be based. We hypothesize that an individualized approach based on comorbidities and clinical history could be more accurate. These findings point toward the necessity of more (prospective) research to support the refinement of postoperative monitoring guidelines to optimize healthcare resource allocation, while maintaining patient safety.

## 1. Introduction

Inguinal hernia (IH) is a common congenital problem among infants, with an incidence of 1 to 5% in term children and up to 20% in preterm infants [1,2]. If left untreated, the patient could be at risk for incarceration. The rate of incarceration is reported to range from 3% to 16% in all children, and it can be as high as 31% in premature infants [1,3,4,5]. Incarceration may lead to bowel ischemia and warrants emergency inguinal hernia repair (IHR), which leads to a higher risk of postoperative complications. The incidence of complications during or after surgery of an incarcerate hernia range can be up to 10%, compared to 1% when IHR is performed in an elective setting “soon” after its diagnosis (some reports suggest even lower complication rates when using a laparoscopic technique) [3,6,7,8,9]. These risks and potential complications justify the need for surgical repair of the IH, usually performed under general anesthesia in our center, combined with a loco-regional block, most often a caudal block [10].

However, anesthesia in infants comes with its own risks. It is established that neonates and infants are more vulnerable to intraoperative and postoperative anesthesia-related complications compared to older children [11]. Other factors that increase this risk are prematurity and a history of respiratory problems [12,13,14]. Possible anesthesia-related complications include postoperative apnea, aspiration, cardiac arrhythmias, and, in some older cases, even cardiac arrest has been reported [15]. Most of such episodes occur in the first 12 h after surgery [16,17]. The current guidelines in most institutions dictate to monitor preterm infants overnight following IHR, whereas in term infants, the surgery is mostly performed as a day care procedure. There is no clear consensus about which (preterm) patients should be monitored after IHR. Most institutions use postconceptional age (PCA) as a cut off for postoperative monitoring, with the threshold set to values ranging from 44 to 60 weeks PCA [18]. This is based on historical reports, where the incidence of these respiratory events in preterm infants was as high as 49% [12,13,14,17,19,20,21]. However, the majority of the more recent literature describes lower rates of anesthesia-related complications in preterm infants, with one study even having no cases at all [16,22,23,24,25,26,27].

Given the lowering trend of postoperative respiratory complications, multiple researchers have put into question the current guidelines on postoperative monitoring of infants and whether a more tailored approach could be possible [22,24,25,28,29,30]. This would be relevant to minimize unnecessary hospital stays and optimize resource utilization. Based on the current protocols for postoperative monitoring, beds in the hospital might be reserved for a patient that potentially do not need them, while another patient that is in need of a hospital bed, e.g., for urgent pathologies, could be sent away. Unfortunately, this is the reality of the ever-growing pressure on the current medical system. Constructing a more precise model of decision making regarding postoperative monitoring in infants, based on other important individual factors, such as respiratory history and other comorbidities, could minimize the overestimation of patients needing to stay overnight. This is important to reassure parents, organize the healthcare more efficiently and reduce unnecessary costs [31].

The aim of this study is to contribute to the existing evidence regarding the detection of postoperative complications from anesthesia after IHR in children under 3 months of age. Furthermore, we aim to identify potential risk factors associated with these postoperative complications.

## 2. Materials and Methods

### 2.1. Patient Inclusion

The Institutional Review Board (or Ethics Committee) of Maastricht University Medical Center+ (MUMC+) approved this study on 17 June 2021 (2021-2774). A retrospective cohort was constructed with all patients under the age of 3 months who underwent inguinal hernia repair in the MUMC+ in the period between November 2015 and August 2023. Given the purely retrospective nature of the research and the anonymization of all data, no informed consent form was necessary or obtained. The only reason for exclusion was a combination of surgeries during one anesthesia that significantly influenced the anesthesia time. IHR combined with, e.g., umbilical hernia repair, was not excluded.

### 2.2. Anesthesia and Surgical Technique

In our facility, all IHR are performed under general anesthesia, most commonly combined with a caudal block or sometimes with an ilio-inguinal block, and different surgical techniques for pediatric inguinal hernia repair are applied: open repair (high ligation), laparoscopic-assisted percutaneous internal ring suturing (PIRS), and conventional laparoscopic inguinal hernia repair (LIHR) or robot-assisted inguinal hernia repair (RIHR). The national guidelines in the Netherlands state that low-risk surgical procedures, like IHR, in children can be performed in a daycare setting if (a) the patient is older than 4 weeks in the case of a term birth, or (b) the patient has a PCA of at least 60 weeks in the case of a premature birth (defined as less than 37 weeks of pregnancy), depending on comorbidities [29]. This guideline is also applied in our facility. In case the patient needs to be monitored overnight, there are different options: intermittent monitoring (normal ward), the Medium Care unit (MC), more frequent monitoring of heart rate, respiratory rate oxygen saturation, electrocardiogram and blood pressure, and the Neonatal or Pediatric Intensive Care unit (NICU/PICU), where patients are continuously monitored. The anesthesiologists decide the postoperative destination of the patient before the surgery. The follow-up was performed in a standard manner, according to our facilities guidelines, with a postoperative control visit 4 weeks after surgery.

### 2.3. Outcomes and Data Collection

The primary outcome was the detection rate of postoperative complications possibly related to anesthesia. Given there is no standard classification tool for possible anesthesia-related events, we scored the events dichotomously, as either present or absent. All deviations from a normal postoperative course were included, whether they were (likely) anesthesia-related or not. Examples of potential anesthesia-related outcomes that could be included are apnea, cardiac arrhythmias, aspiration and fever. The secondary outcome was the detection rate of events that were ‘likely’ related to anesthesia, where events that could clearly be traced back to another underlying cause were excluded. To obtain all information about events, the entire electronic patient file was consistently reviewed, since events might be recorded in different manners; in (NICU/PICU) charts, nurses notes, recovery notes, monitoring alarms, documentation by a physician or other medical staff, discharge letters, etc. In case an event was recorded in any of these modalities, it was recorded in our database as being ‘present’, regardless of the necessity for intervention or the intensity of the event.

The events were scored according to the Clavien-Madadi classification, as there is currently no specific classification for anesthesia-related complications in children and the Clavien-Madadi classification takes every ‘unexpected event’ into account. This is defined as ‘any event with subsequent deviations from the planned, pre-, intra-, and postoperative courses of patients’, and thus is not only limited to complications related to the surgical procedure itself, but events related to the whole process [32]. Other variables collected included patient demographics (antenatal and perinatal data, sex, weight, comorbidities, and ASA classification), surgical data (procedure, surgery setting, and anesthetic management) and post-operative outcomes (perioperative monitoring, complications and length of stay). The PCA at surgery was calculated for all patients as GA plus actual age in weeks. The surgery setting was classified as either elective, emergency or semi-acute, with the latter being that a patient presented at the ER with an incarcerated hernia, which was reduced, after which the patient was admitted and underwent surgery the next day. The data were extracted from the electronic medical records of all eligible patients. Data extraction was performed manually, utilizing a standardized form to ensure accuracy.

### 2.4. Statistical Analysis

Patients were divided into two groups, based on whether they experienced possible anesthesia-related complications within the first 24 h after surgery. Baseline characteristics and all outcomes were presented using the mean and standard deviation (SD) for continuous variables. Discrete variables were presented using absolute numbers (*n*) and percentages (%). Variables were tested for normality using the Kolmogorov–Smirnov test and the histograms and Q-Q plots were visually inspected. To compare the baseline characteristics across the two groups for continuous variables, a *t*-test was performed in case of normality; otherwise, the Mann–Whitney U test was carried out. In case of categorical variables, the Chi square test was performed to test for significant differences.

A multivariable-adjusted logistic regression model was not possible, because of the low number of events. A univariate logistic regression analysis was performed with PCA at surgery and GA, as these are the variables that are mentioned in most current protocols and guidelines as being risk factors for anesthesia-related events after surgery.

Tests were considered statistically significant if *p* < 0.05. For the statistical analysis, the IBM SPSS Statistics for Windows, version 28.0 (IBM Corp., Armonk, NY, USA) software was used.

## 3. Results

### 3.1. Population Characteristics

In total, the medical records of 320 patients were reviewed. Two patients were excluded due to combination surgery (one IHR combined with liver biopsy and cholangiography, one IHR combined with laser treatment for retinopathy of prematurity (ROP)) and twelve were duplicates of patients undergoing IHR for a recurrent hernia in the same period. For these patients, the records of the first surgery were included. In total, 306 patients were included that underwent IHR in our institution while three months old or younger.

The majority of patients were male (85.9%) and had a gestational age of more than 37 weeks (63.7%). The mean age at surgery was 11.2 ± 4.4 weeks, with a mean PCA of 47.7 ± 4.8 weeks. All patient characteristics are shown in Table 1.

When comparing the group with possible anesthesia-related complications within 24 h after surgery with the group without complications, there was no significant difference in GA (*p* = 0.539) or PCA (*p* = 0.451). The only characteristics that showed a significant difference between both groups were a history of respiratory problems (7.7% in the group without complications vs. 30.0% in the group with complications, *p* = 0.013) and circulatory problems (8.1% in the group without complications vs. 30.0% in the group with complications, *p* = 0.016).

When comparing the decided monitoring necessity for the patients and the eventual anesthesia-related complications, there was no significant difference between the monitoring status of the patients who had complications and those who did not (*p* = 0.512).

Most of the procedures were performed in an elective setting (79.1%), with no significant difference between the groups (*p* = 0.168). Open herniotomy was the most common technique (68.4%), compared to the laparoscopic techniques (PIRS 16.8%, LIHR 14.8%). Eleven subjects (3.6%) experienced anesthesia-related intraoperative complications (apnea, bronchospasms or laryngospasms, bradycardia, hypotension), nine of which needed some form of intervention. Consequently, the total anesthesia time was also significantly different between both groups (*p* = 0.047). Three per-operative complications occurred in the same patients that also developed anesthesia-related postoperative complications. All per-operative characteristics are reported in Table 2.

### 3.2. Primary Outcome Measure: Possible Anesthesia-Related Complications

Of the 306 included patients, ten patients (3.3%) experienced (possible) anesthesia-related complications within the first 24 h after surgery. Of the (possible) anesthesia-related complications, six occurred while the patient was still in the post-anesthesia care unit (PACU). Six events were observed on the ward; two patients experienced an event in the PACU and on the ward. Events included desaturations, tachycardia, convulsions, fever and a choking incident. Intervention, such as administration of extra O_2_, was necessary in six of ten patients.

When comparing the detection rate of anesthesia-related events in prematurely born children (*n* = 4, 3.6%) and term born children (*n* = 6, 3.1%), the difference was not significant (*p* = 0.719). According to the Clavien-Madadi classification, eight patients experienced complications that were grade IB and two were grade II [32]. Table 3 shows an overview of the characteristics of the subjects that experienced anesthesia-related complications, including details about the event, necessary interventions and the Clavien-Madadi classification.

As can be expected, subjects who did have complications had a significantly longer hospital stay (3.1 ± 1.1 days vs. 1.0 ± 0.1 days, *p* < 0.001). No anesthesia-related complications occurred after 24 h post-operation and there were no readmissions due to anesthesia-related complications.

### 3.3. Secondary Outcome Measure: Likely Anesthesia-Related Complications

In case 9, the persistent fever was deemed unlikely to be anesthesia-related, as the patient presented with fever upon arrival at the emergency department. The underlying cause was identified as incarcerated adnexa. Per-operatively, antibiotics were initiated and continued postoperatively until the patient was afebrile and could be discharged home. In case 10, the postoperative evaluation revealed a previously undiagnosed large ventricular septal defect (VSD) and atrial septal defect (ASD), which were considered the likely underlying cause of the patient’s postoperative symptoms. Consequently, within this case series, only eight patients (2.6%) were thought to have suffered likely anesthesia-related complications.

### 3.4. Correlation Between PCA or GA and Postoperative Anesthesia-Related Events

Two univariate logistical regression models were created with possible anesthesia-related postoperative events within 24 h after surgery as the dependent variable. The non-dependent variables were GA and PCA at surgery.

The model with GA as a predictor variable was not statistically significant, χ^2^(1) = 2.2, *p* = 0.528. The model explained 3% of the variance in the outcome variable (Nagelkerke R^2^ = 0.031). When modeling PCA at surgery as a predictor variable in a logistic regression model, this model was also not significant, χ^2^(1) = 0.7, *p* = 0.414. The model explained 0.9% of the variance in the outcome (Nagelkerke R^2^ = 0.009).

This suggests that there was no strong, individual association between gestational age or PCA at surgery and anesthesia-related complications within 24 h after surgery. The odds ratio (OR) for anesthesia-related complications within 24 h after surgery was also not significant for a GA < 37 weeks, OR = 1.138 (95% CI = [0.279; 4.644]).

### 3.5. Number Needed to Monitor (to Detect One Additional Event)

As the current guidelines to decide the postoperative monitoring protocol are based on whether the patient was born prematurely or not, we looked at the detection rate of likely anesthesia-related complications (secondary outcome measure) in these two groups to calculate the number needed to monitor to detect one additional event, based on these protocols (NNM). In the group of prematurely born children (<37 weeks GA), the detection rate was 2.70% (*n* = 3) in our cohort. For the group that was born at term, the detection rate was 2.56% (*n* = 5). With these detection rates, the absolute risk reduction (ARR) is 0.0014 (95% CI = [0.00139; 0.00139], meaning the NNM in order to detect one additional event would be 721 patients (95% CI = [721.46206; 721.53794]).

## 4. Discussion

This retrospective cohort study aimed to evaluate the detection rate of possible anesthesia-related postoperative complications in patients who underwent inguinal hernia repair at the age of three months or younger. Furthermore, potential risk factors for possible anesthesia-related events were explored through analysis of patient characteristics.

The overall detection rate of potential anesthesia-related events after IHR was relatively low (3.3%, *n* = 10) in our population, which is in line with the more recent literature, where incidences are reported between 0% and 7% [16,23,24,26,28,29]. Historically, rates for postoperative respiratory complications, such as apnea, a need for postoperative assisted ventilation or even reintubation, have been reported to be as high as 43% in premature infants [12,13,19,20,21]. After the first report in 1982 (Steward et al., 1982), most centers set into place a protocol to monitor preterm infants postoperatively [13,14,18,29,33,34]. The current guideline by the American Academy of Pediatrics (AAP), for example, states that ‘admission and monitoring should be planned for at least 12 h after anesthesia and surgery for preterm infants younger than 50 to 60 weeks PCA and full-term infants younger than 4 weeks’ [35]. However, more recent studies suggest that the rate of postoperative apnea is much lower in preterm infants [16,22,23,24,25,26,27]. A possible explanation for this large range in incidence is the fact that respiratory monitoring is not standardized and depends on the type of monitor that is used, with a doubling of occurrence when continuous monitoring is used [14,16,34]. Furthermore, it is hard to compare the literature, as there is no uniform definition or classification of apnea and respiratory events. Even so, there seems to be general decline in respiratory problems following inguinal hernia repair with general anesthesia. This could be due to multiple factors, including development and advancement in anesthetic agents and techniques, improved postoperative care and better risk stratification [17,18].

All these findings put into question the current protocols and guidelines on postoperative monitoring of, especially, ex-prematurely born infants. Our data did not find a significant difference in the incidence of possible anesthesia-related events between prematurely born children compared to term-born children (3.6% vs. 3.1% respectively, *p* = 0.719). When looking at the likely anesthesia-related events, we found a number needed to monitor to detect one additional event of 721 patients. As this is based solely on prematurity, this is of course not a metric to be used as a direct guide for clinical decision making, but it merely illustrates that there might be a discrepancy between the current guidelines and the clinical reality.

Of course, monitoring is noninvasive and could prevent bodily harm, so an NNM of 721 would not be put into question. However, many of the events that are recorded through monitoring are not life-threatening and resolve spontaneously, without the need for any intervention. In our population, four of the ten patients experiencing an event did not require any treatment. This raises the question of if it is necessary to even observe all these events. There was also no significant difference in anesthesia-related events between the patients who underwent (any kind of) monitoring postoperatively, compared to those who were kept on the regular ward or in the outpatient clinic (2.1% vs. 4.4% respectively, *p* = 0.827), indicating that the current protocol might not cover all patients who are at risk of an incident. Otherwise, significantly more events would be seen in the groups that are more intensely monitored, even if these events might not be clinically relevant. This was not the case and a significant number of events still happened during their stay on the regular ward, without intensive monitoring. These facts illustrate that current protocols for postoperative monitoring might not be tailored to the population that effectively needs it to prevent life-threatening anesthesia-related complications.

This issue has been addressed previously by other researchers. Many studies have tried to identify risk factors for anesthesia-related complications to improve the selection of patients who need postoperative monitoring [12,13,14,16,17,18,19,20,21,22,23,24,25,26,27,28,29,30,34]. The most reported risk factors that are described are a history of respiratory problems and/or a low PCA, with a cut off for increased risk at around 45 weeks PCA in most studies [12,13,14,19,20,25,28,29,30]. In our population, we did not find a significant difference when comparing the groups with or without anesthesia-related complications in terms of GA, PCA, actual age or birth weight (factors that are often identified as risk factors in other studies). There was a significant difference between both groups when looking at respiratory or circulatory comorbidities (*p* = 0.013 and *p* = 0.016 respectively).

Our findings support the growing body of literature suggesting that routine overnight monitoring for ex-premature infants may not be necessary for all, especially in the absence of other risk factors. We corroborate that a multifactorial risk assessment should be made, considering medical history and comorbidities in particular, instead of only PCA as a single criterion for the decision-making. It is important to target the patients who effectively need postoperative monitoring and not to overestimate the use for monitoring, because there is an ever-growing pressure on the healthcare system. Reducing the need for infants staying overnight in the hospital will have benefits on more than one level. Parents will be more reassured about the condition of their child and unnecessary costs for unnecessary monitoring will be avoided. The most important reason, however, is the optimization of healthcare resource allocation [31]. By monitoring patients who might not be at risk, a bed in the ward is occupied, and in turn cannot be used for another patient who might be in need of urgent care.

This study has multiple limitations. The biggest limitation is the retrospective nature of the data collection, creating the possibility for bias in the outcomes. One area where there might be bias is the fact that postoperative monitoring can take on many forms, so all patients are monitored in a different way. Some are continuously monitored overnight postoperatively, while others were only continuously monitored in the PACU, and were observed on the ward afterwards (without continuous monitoring). This is a limitation across several studies, especially when comparing results, as there are no specific guidelines on what sort of monitoring should be implemented when and what is considered a significant event. However, we believe our data makes a valuable contribution to the existing literature concerning the topic of postoperative monitoring after IHR in children, as we describe in depth multiple parameters and outcomes of 306 consecutive IHRs in a university medical center.

In the future, a large, multicenter trial (with standardized methods of monitoring) would aid in the evaluation of the current guidelines for postoperative monitoring in infants and to identify which patients, with what risk factors, truly benefit from intensified postoperative monitoring. For now, we would recommend a tailored approach in the decision-making concerning the need for postoperative monitoring, with a close look at each patient’s individual risk factors and the sound clinical judgment of the anesthesiologist. In the future, decisions based solely on PCA might be adapted.

## 5. Conclusions

In our study population, the detection rate of (potential) anesthesia-related complications after inguinal hernia repair in infants under three months of age was low. Our results corroborate multiple studies suggesting that prematurity and PCA alone may not justify routine postoperative monitoring. Our data give rise to the hypothesis that individual risk factors, such as medical history and comorbidities, may offer a more accurate and selective approach in the decision making process, optimizing healthcare resource allocation while maintaining patient safety. Of course, these data are retrospective and heterogeneous, so a large, prospective, multicenter study is necessary to draw accurate conclusions to aid in the adaptation of the current guidelines and protocols.

## Figures and Tables

**Table 1:** Baseline characteristics of total study population and of the population divided by whether they experienced anesthesia-related complications within 24 h after surgery or not.

	Total (*n* = 306)	Patients Without Anesthesia-Related Complication < 24 h Postoperatively (*n* = 296)	Patients with Anesthesia-Related Complication < 24 h Postoperatively (*n* = 10)	*p*-Value
**Sex** Male (%)	263 (85.9)	255 (86.1)	8 (80.0)	0.582
**Birth Weight** (kg) Mean ± SD	2.6 ± 0.9	2.6 ± 00.9	2.8 ± 1.1	0.648
**Gestational Period** *n* (%)				0.539
>37 weeks	195 (63.7)	189 (63.9)	6 (60.0)	
36–33 weeks	72 (23.5)	70 (23.6)	2 (20.0)	
32–28 weeks	31 (10.1)	29 (9.8)	2 (20.0)	
<27 weeks	8 (2.6)	8 (2.7)	0 (0.0)	
**Weight at surgery** (kg) Mean ± SD	4.5 ± 1.1	4.5 ± 1.1	4.2 ± 0.9	0.411
**(Actual) Age at surgery** (weeks) Mean ± SD	11.2 ± 4.4	11.2 ± 4.4	9.9 ± 4.7	0.352
**PCA at surgery** (weeks) Mean ± SD	47.7 ± 4.8	47.8 ± 4.9	46.6 ± 2.3	0.451
**Medical history** *n* (%)				
Twin	26 (8.5)	26 (8.8)	0 (0.0)	0.327
Fetal distress/Emergency C-section	28 (9.2)	26 (8.8)	2 (20.0)	0.226
Dysmaturity/IUGR/SGA	41 (13.4)	39 (13.2)	2 (20.0)	0.788
Respiratory	26 (8.5)	23 (7.7)	3 (30.0)	0.013 *
Circulatory	27 (8.8)	24 (8.1)	3 (30.0)	0.016 *
Neurological	9 (2.9)	9 (3.0)	0 (0.0)	0.576
Infectious	18 (5.9)	16 (5.4)	2 (20.0)	0.054
Other	58 (19.0)	57 (19.3)	1 (0.1)	0.463
Prior surgery	4 (1.3)	4 (1.4)	0 (0.0)	0.711
**Concomitant medication ^a^** *n* (%)	40 (13.1)	37 (12.5)	3 (30.0)	0.106
**Unilateral Diagnosis** *n* (%)	230 (75.2)	224 (75.7)	6 (60.0)	0.259
**Strangulation** *n* (%)				0.198
Reducible	44 (14.4)	42 (14.2)	2 (20.0)	
Non-reducible	20 (6.5)	18 (6.1)	2 (20.0)	
**ASA Classification** *n* (%)				0.404
ASA 1	217 (70.9)	212 (71.6)	5 (50.0)	
ASA 2	75 (24.5)	72 (24.3)	3 (30.0)	
ASA 3	12 (3.9)	11 (3.7)	1 (10.0)	
**Postoperative monitoring** *n* (%)				0.512
No monitoring (outpatient clinic)	161 (52.6)	154 (52.0)	7 (70.0)	
MC	139 (45.4)	136 (45.9)	3 (30.0)	
NICU/PICU	6 (2.0)	6 (2.0)	0 (0.0)	

Kolmogorov–Smirnov test was used to test continuous variables for normality. In case of normal distribution, *t*-test was performed, otherwise Mann–Whitney U was used. For categorical variables, Chi^2^ test was used to compare variables. * indicates statistical significance, α = 0.05. ^a^ Concomitant medication included ferrous fumarate, antacids, laxatives, antibiotics or a combination thereof. Abbreviations: PCA, postconceptional age; IUGR, intrauterine growth restriction; SGA, small for gestational age; ASA, American Society of Anesthesiologists; MC, Medium Care; NICU, Neonatal Intensive Care Unit; and PICU, Pediatric Intensive Care Unit.

**Table 2:** Per-operative characteristics of the total study population and of the population divided by whether they experienced anesthesia-related complications within 24 h after surgery or not.

	Total (*n* = 306)	Patients Without Anesthesia-Related Complication < 24 h Postoperatively (*n* = 296)	Patients with Anesthesia-Related Complication < 24 h Postoperatively (*n* = 10)	*p*-Value
**Surgery setting** *n* (%)				0.168
Elective	242 (79.1)	236 (79.7)	6 (60.0)	
Semi-acute (ER visit, surgery next day)	44 (12.4)	42 (14.2)	2 (20.0)	
Acute (ER visit, surgery same day)	20 (6.5)	18 (6.1)	2 (20.0)	
**Surgical technique** *n* (%)				0.090
Open herniotomy	204 (66.7)	198 (66.9)	6 (60.0)	
PIRS	51 (16.8)	47 (15.9)	4 (40.0)	
LIHR	44 (14.4)	44 (14.9)	0 (0.0)	
**Block anesthesia** *n* (%)				0.404
None	45.0 (14.7)	44 (14.9)	1 (10.0)	
Caudal	252 (82.4)	244 (82.4)	8 (80.0)	
Ilio-inguinal	7 (2.3)	6 (2.0)	1 (10.0)	
Caudal and ilio-inguinal	1 (0.3)	1 (0.3)	0 (0.0)	
**Anesthesia time** (min) Mean ± SD	82.8 ± 1.7	82.2 ± 29.1	101.0 + 35.1	0.047 *
**Net surgical time** (min) Mean ± SD	37.5 ± 1.3	37.2 ± 21.7	45.9 ± 23.9	0.216
**Per-operative complications** *n* (%)				<0.001 *
None	290 (94.8)	283 (95.6)	7 (70.0)	<0.001 *
Respiratory	7 (2.3)	5 (1.7)	2 (20.0)	0.003 *
Circulatory	2 (0.7)	2 (1.7)	0 (0.0)	0.993
Surgical	2 (0.7)	1 (0.3)	1 (10.0)	0.003 *

Kolmogorov–Smirnov test was used to test continuous variables for normality. In case of normal distribution, *t*-test was performed, otherwise Mann–Whitney U was used. For categorical variables, Chi^2^ test was used to compare variables. * indicates statistical significance, α = 0.05. Abbreviations: ER, emergency room; PIRS, percutaneous internal ring suturing; LIHR, (conventional) laparoscopic inguinal hernia repair.

**Table 3:** Characteristics of the subjects that experienced anesthesia-related postoperative events within the first 24 h after inguinal hernia repair.

Case	1	2	3	4	5	6	7	8	9	10
**Sex**	M	M	M	M	M	M	F	F	F	M
**Birth Weight** (kg)	-	1.4	3.3	2.8	4.5	3.4	3.2	3.5	-	1.9
**Gestational Age** (weeks)	40	29	39	33	41	39	40	38	30	37
**Age at Surgery** (weeks)	8	16	7	12	4	6	5	14	17	10
**PCA** (weeks)	48	45	46	45	5	45	45	52	47	47
**Weight at Surgery** (kg)	4.3	3.8	4.6	4.5	5	4.1	3.5	6	5.1	3
**Medical History**	**-**	BPD, NEC, stoma, cholestasis due to TPN, self-limiting saturation drops and bradycardia requiring cardiorespiratory support	-	PPS	-	PPROM	-	-	CPAP support	Large VSD and ASD with hemodynamic significance (diagnosed postoperatively)
**Concomitant Medication**	-	Nystatin, Vitamin D, Miconazole, Ferrous fumarate, Propranolol	-	Ferrous fumarate	-	-	Omeprazole	Vitamin D and K	-	-
**Diagnosis**	IH R	IH Bi	IH R	IH L + UH	IH R	IH R	IH L + UH	IH R	IH R	IH Bi
**Strangulation**	Reducible	Non-reducible	Reducible	-	-	Non-reducible	-	-	Non-reducible	-
**Surgery Setting**	Semi-acute	Emergency	Semi-Acute	Elective	Elective	Emergency	Elective	Elective	Emergency	Elective
**ASA** **Classification**	1	3	1	2	1	1	1	1	2	2
**Surgical** **Technique**	PIRS	Open	Open	PIRS	PIRS	Open	Open	Open	Open	PIRS
**Block Anesthesia**	Caudal	Caudal	Caudal	Caudal	Caudal	Caudal	Caudal	Local	Caudal	None
**Anesthesia Time** (min)	129	179	77	69	108	75	79	74	89	126
**Net Surgical Time** (min)	79	72	33	63	70	13	31	29	64	50
**Per-operative complications**	-	-	-	-	Bronchospasm with hypoxemia to 19%, no bradycardia, just after extubation → restart ventilation	-	-	-	-	-
**Complications of PACU**	Desaturation with cyanosis and bradycardia → Resolved after O_2_	Respiratory and circulatory insufficiency → Spontaneous recovery	Tachycardia and hypertension → Spontaneous recovery	Tachycardia, remarkable blue tongue → Spontaneous recovery	Desaturation → Resolved after O_2_ administration	Desaturation → Resolved after O_2_ administration	-	-	-	-
**Complications on ward**	-	-	-	-	Desaturation → Spontaneous recovery	Desaturation → Resolved after O_2_ administration	Choking incident at night → no intervention	Convulsions → 0.5 mg Midazolam	Persistent fever → IV augmentin	Desaturation to 70%, tachydyspnoeic → Resolved after O_2_. Start diuretics and beta blockers
**Clavien-Madadi classification**	IB	IB	IB	IB	IB	IB	IB	II	II	IB

Abbreviations: PCA, postconceptional age; BPD, bronchopulmonary dysplasia; NEC, necrotizing enterocolitis; TPN, total parenteral nutrition; PPS, peripheral pulmonic stenosis; PPROM, prolonged preterm premature rupture of membranes; CPAP, continuous positive airway pressure; VSD, ventricular septal defect; ASD, atrial septal defect; IH R, right-sided inguinal hernia; IH Bi, bilateral inguinal hernia; IH L, left-sided inguinal hernia; UH, umbilical hernia; ASA, American Society of Anesthesiologists; PIRS, percutaneous internal ring suturing; PACU, Post-Anesthesia Care Unit.

## Data Availability

The data presented in this study are available upon request from the corresponding author, due to the privacy of the included subjects.

## Abbreviations

The following abbreviations are used in this manuscript:

GA	Gestational Age
IH	Inguinal Hernia
IHR	Inguinal Hernia Repair
LIHR	Laparoscopic Inguinal Hernia Repair
MC	Medium Care Unit
NICU	Neonatal Intensive Care Unit
PACU	Post-Anesthesia Care Unit
PCA	Postconceptional Age
PICU	Pediatric Intensive Care Unit
PIRS	Percutaneous Internal Ring Suturing
UH	Umbilical Hernia
